# Supplementary material for: Microbial Community Shifts in Tea Plant Rhizosphere under Seawater Stress: Enrichment of Beneficial Taxa
Source: Microorganisms. 2024 Jun 25;12(7):1287. doi: 10.3390/microorganisms12071287 (PMC11279268; doi:10.3390/microorganisms12071287)
Supplement: Supplementary file 1 [file microorganisms-12-01287-s001.zip › microorganisms-3035812-supplementary.pdf]

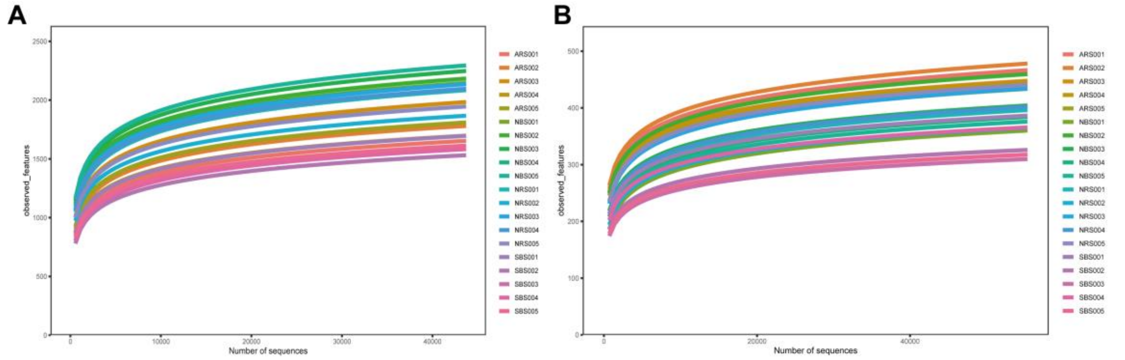

Figure S1 Rarefaction curve of soil bacteria (A) and fungus (B).

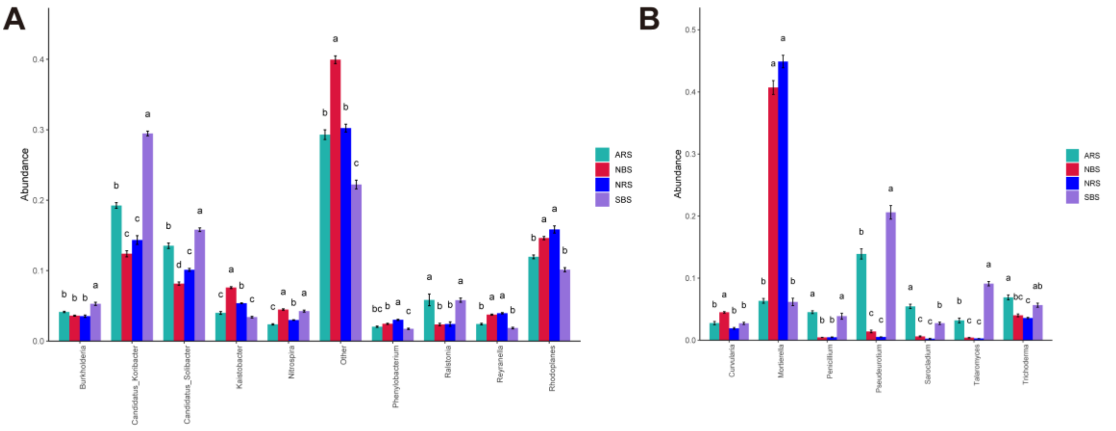

Figure S2 Multiple comparisons of the top ten genera in terms of relative abundance of bacteria (A) and fungi (B) using ANOVA analysis ($p<0.05$).

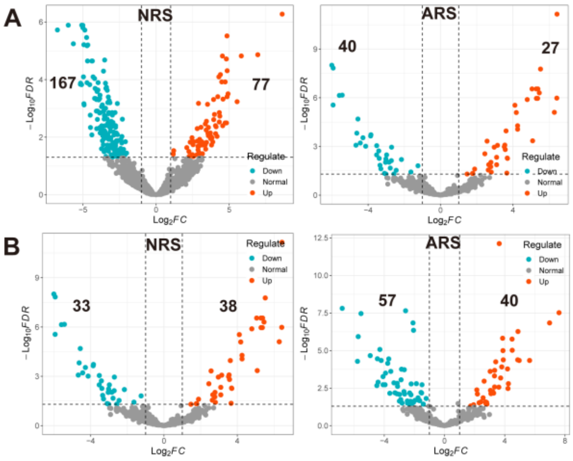

Figure S3 The volcano plot illustrating the enrichment and depletion patterns of rhizosphere bacterial (A) and fungal (B) microbiomes in the control and seawater stressed samples. Each point represents a ASV. Each red point represents an individual enriched ASV, and a green point represents an individual depleted ASV.

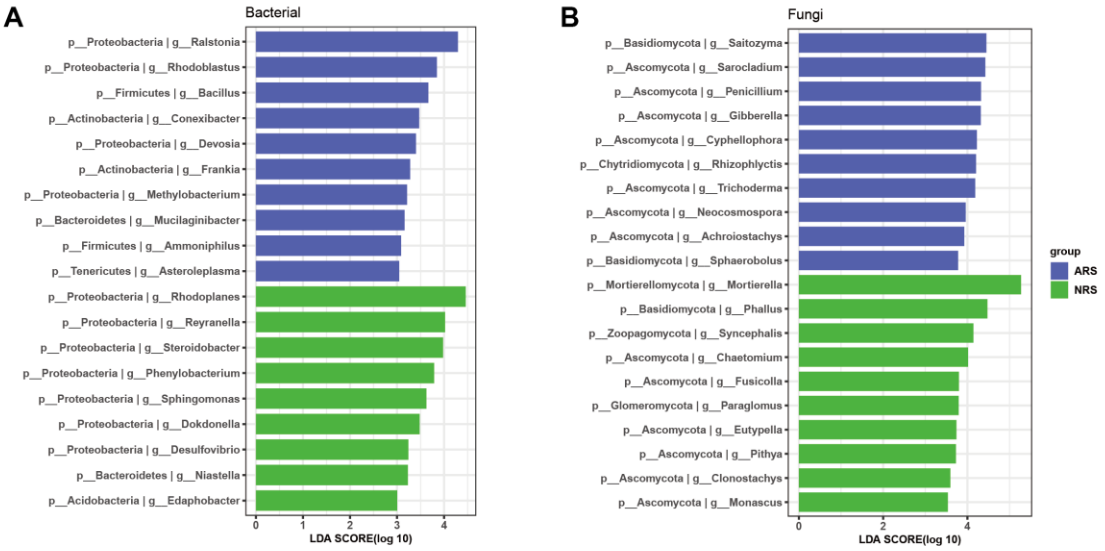

Figure S4 Linear discriminant analysis (LDA) of effect Size (LEfSe) of the top 10 bacterial (A) and fungi (B) taxa enriched in the rhizosphere of tea plant.

Table S1 Statistical table of the process of bacterial dada2 denoising to generate ASVs.

sample-id	input	filtered	percentage of input passed filter	denoised	merged	percentage of input merged	non-chimeric	percentage of input non-chimeric
ARS001	84,260.0	77,673.0	92.18	73,584.0	57,475.0	68.21	52,145.0	61.89
ARS002	88,012.0	80,773.0	91.77	76,192.0	60,457.0	68.69	55,328.0	62.86
ARS003	87,906.0	81,557.0	92.78	76,837.0	59,990.0	68.24	54,213.0	61.67
ARS004	81,908.0	75,279.0	91.91	70,330.0	51,782.0	63.22	46,722.0	57.04
ARS005	87,384.0	79,799.0	91.32	74,975.0	58,823.0	67.32	53,474.0	61.19
NBS001	83,221.0	76,340.0	91.73	71,217.0	53,983.0	64.87	47,147.0	56.65
NBS002	79,260.0	73,463.0	92.69	68,500.0	51,281.0	64.70	44,272.0	55.86
NBS003	84,844.0	78,055.0	92.00	73,075.0	55,805.0	65.77	48,856.0	57.58
NBS004	78,957.0	72,643.0	92.00	67,741.0	51,667.0	65.44	45,261.0	57.32
NBS005	83,845.0	77,785.0	92.77	72,717.0	56,102.0	66.91	49,258.0	58.75
NRS001	89,884.0	82,883.0	92.21	77,856.0	58,545.0	65.13	51,514.0	57.31
NRS002	78,671.0	72,023.0	91.55	67,651.0	52,572.0	66.83	45,602.0	57.97
NRS003	89,560.0	82,956.0	92.63	77,817.0	60,114.0	67.12	53,203.0	59.40
NRS004	86,463.0	79,368.0	91.79	74,372.0	57,286.0	66.25	50,422.0	58.32
NRS005	84,412.0	78,508.0	93.01	75,157.0	62,768.0	74.36	55,650.0	65.93
SBS001	85,910.0	78,643.0	91.54	73,963.0	56,819.0	66.14	51,406.0	59.84
SBS002	88,959.0	81,147.0	91.22	76,760.0	59,146.0	66.49	53,770.0	60.44
SBS003	83,804.0	76,831.0	91.68	72,719.0	57,791.0	68.96	52,816.0	63.02
SBS004	84,001.0	76,177.0	90.69	71,985.0	54,963.0	65.43	49,791.0	59.27

Table S2 Statistical table of the process of fungi dada2 denoising to generate ASVs.

sample-id	input	filtered	percentage of input passed filter	denoised	merged	percentage of input merged	non-chimeric	percentage of input non-chimeric
ARS001	87,546.0	79,768.0	91.12	78,634.0	76,557.0	87.45	75,765.0	86.54
ARS002	81,939.0	75,492.0	92.13	74,312.0	72,427.0	88.39	72,000.0	87.87
ARS003	80,139.0	73,488.0	91.70	72,497.0	70,754.0	88.29	70,149.0	87.53
ARS004	80,992.0	74,435.0	91.90	73,439.0	71,306.0	88.04	70,541.0	87.10
ARS005	82,790.0	76,223.0	92.07	75,176.0	73,249.0	88.48	72,609.0	87.70
NBS001	87,679.0	80,963.0	92.34	79,994.0	78,475.0	89.50	77,685.0	88.60
NBS002	88,392.0	81,028.0	91.67	79,612.0	77,273.0	87.42	76,218.0	86.23
NBS003	87,674.0	80,120.0	91.38	79,013.0	76,687.0	87.47	75,956.0	86.63
NBS004	83,167.0	76,334.0	91.78	75,047.0	71,838.0	86.38	70,755.0	85.08
NBS005	89,756.0	81,606.0	90.92	80,508.0	78,523.0	87.48	77,443.0	86.28
NRS001	91,795.0	84,300.0	91.84	83,204.0	81,608.0	88.90	80,924.0	88.16
NRS002	82,851.0	75,969.0	91.69	74,989.0	73,260.0	88.42	72,643.0	87.68
NRS003	82,817.0	75,912.0	91.66	74,803.0	72,980.0	88.12	71,873.0	86.79
NRS004	84,506.0	77,633.0	91.87	76,672.0	74,676.0	88.37	74,032.0	87.61
NRS005	88,677.0	80,794.0	91.11	79,493.0	77,175.0	87.03	76,314.0	86.06
SBS001	91,465.0	84,221.0	92.08	83,225.0	81,082.0	88.65	80,203.0	87.69
SBS002	78,684.0	73,061.0	92.85	72,388.0	71,058.0	90.31	69,530.0	88.37
SBS003	84,420.0	77,975.0	92.37	77,249.0	75,566.0	89.51	74,892.0	88.71
SBS004	74,015.0	68,244.0	92.20	67,391.0	66,130.0	89.35	65,283.0	88.20

Table S3 Effects of seawater intrusion on the changes of bacterial, fungal composition based on PERMANOVA.

Type		Bacterial community	Fungi community
Rhizosphere (ARSvsNRS)	P	$P<0.05$	$P<0.05$
	R^2	0.48	0.78
Bulk soil (SBSvsNBS)	P	$P<0.05$	$P<0.05$
	R^2	0.65	0.76
Normal site (NBSvsNRS)	P	$P<0.05$	$P<0.05$
	R^2	0.33	0.40
Stressed site (SBSvsARS)	P	$P<0.05$	$P<0.05$
	R^2	0.32	0.46

Table S4 Details of hub tax in NRS co-occurrence network.

kingdom	phylum	Genus	Degree	Abundance	Closeness Centrality	Harmonic Closeness Centrality	Betweenness Centrality
Bacteria	Proteobacteria	Microvirgula	67	9.8	0.574780059	0.657312925	0.002904946
Bacteria	Proteobacteria	Delftia	67	7.6	0.574780059	0.657312925	0.002904946
Bacteria	Proteobacteria	Agrobacterium	67	5.8	0.574780059	0.657312925	0.002904946
Bacteria	Proteobacteria	Janthinobacterium	67	5.4	0.574780059	0.657312925	0.002904946
Bacteria	Actinobacteria	Cryocola	67	4.2	0.574780059	0.657312925	0.002904946
Bacteria	Proteobacteria	Aquitalea	67	4	0.574780059	0.657312925	0.002904946
Bacteria	Actinobacteria	Solirubrobacter	67	3.6	0.574780059	0.657312925	0.002904946
Bacteria	Bacteroidetes	Myroides	67	3.2	0.574780059	0.657312925	0.002904946
Bacteria	Proteobacteria	Stenotrophomonas	67	3.2	0.574780059	0.657312925	0.002904946
Bacteria	Proteobacteria	Comamonas	67	3	0.574780059	0.657312925	0.002904946
Bacteria	Proteobacteria	Microvirgula	67	9.8	0.574780059	0.657312925	0.002904946
Fungi	Ascomycota	Metacordyceps	74	11.6	0.582656	0.662016	0.012241
Fungi	Ascomycota	Scheffersomyces	74	8.2	0.582656	0.662016	0.012241
Fungi	Ascomycota	Phaeosphaeria	71	104.4	0.540201	0.634884	0.014732
Fungi	Ascomycota	Orbilia	70	679.6	0.548469	0.637984	0.009534
Fungi	Ascomycota	Stagonospora	69	19.6	0.564304	0.644961	0.01337
Fungi	Ascomycota	Oidiodendron	67	224.4	0.523114	0.618605	0.007814
Fungi	Ascomycota	Scytalidium	67	34.6	0.528256	0.621705	0.007887
Fungi	Ascomycota	Issatchenkia	67	8.2	0.523114	0.618605	0.007814

Table S5 Details of hub tax in ARS co-occurrence network.

kingdom	phylum	Genus	Degree	Abundance	Closeness Centrality	Harmonic Closeness Centrality	Betweenness Centrality
Bacteria	Proteobacteria	Mesorhizobium	70	67.6	0.570224719	0.655993432	0.008582427
Bacteria	Bacteroidetes	Hymenobacter	70	4	0.570224719	0.655993432	0.008582427
Bacteria	Actinobacteria	Kutzneria	69	18.4	0.547169811	0.642036125	0.011160477
Bacteria	Proteobacteria	Achromobacter	69	14.2	0.547169811	0.642036125	0.011160477
Bacteria	Actinobacteria	Geodermatophilus	69	10.2	0.547169811	0.642036125	0.011160477
Bacteria	Firmicutes	Listeria	68	39.4	0.578347578	0.65681445	0.003315467
Bacteria	Proteobacteria	Microvirgula	68	14	0.578347578	0.65681445	0.003315467
Bacteria	Bacteroidetes	YRC22	68	12.2	0.578347578	0.65681445	0.003315467
Bacteria	Fibrobacteres	Fibrobacter	68	8.8	0.578347578	0.65681445	0.003315467
Bacteria	Spirochaetes	Treponema	68	6.8	0.578347578	0.65681445	0.003315467
Bacteria	Proteobacteria	Mesorhizobium	70	67.6	0.570224719	0.655993432	0.008582427
Bacteria	Bacteroidetes	Hymenobacter	70	4	0.570224719	0.655993432	0.008582427
Fungi	Ascomycota	Achroiostachys	74	2	0.56927	0.649705	0.006174
Fungi	Basidiomycota	Buckleyzyma	74	2	0.56927	0.649705	0.006174
Fungi	Ascomycota	Ochroconis	74	1.4	0.56927	0.649705	0.006174
Fungi	Ascomycota	Stemphylium	74	1.2	0.56927	0.649705	0.006174
Fungi	Ascomycota	Cercospora	74	1	0.56927	0.649705	0.006174
Fungi	Ascomycota	Zasmidium	74	1	0.56927	0.649705	0.006174
Fungi	Ascomycota	Ramichloridium	74	0.6	0.56927	0.649705	0.006174
Fungi	Ascomycota	Lophiotrema	74	0.6	0.56927	0.649705	0.006174
